# Dishevelled-3 C-terminal His single amino acid repeats are obligate for Wnt5a activation of non-canonical signaling

**DOI:** 10.1186/1750-2187-5-19

**Published:** 2010-11-23

**Authors:** Li Ma, Ying Wang, Craig C Malbon, Hsien-yu Wang

**Affiliations:** 1 Departments of Physiology & Biophysics, Health Sciences Center, State University of New York at Stony Brook, Stony Brook, NY 11794; 2 Department of Oral Biology and Pathology, School of Dental Medicine, State University of New York at Stony Brook, Stony Brook, NY 11794-8702; 3 Department of Pharmacology, Health Sciences Center, State University of New York at Stony Brook, Stony Brook, NY 11794

## Abstract

**Background:**

The Wnt non-canonical pathway (Wnt5a > Frizzled-2 > cyclic GMP phosphodiesterase/Ca^2+^-mobilization pathway regulates the activation of NF-AT) is mediated by three mammalian Dishevelleds (Dvl1, Dvl2, and Dvl3) and the role of the C-terminal region unique to Dvl3 was interrogated.

**Results:**

Dvl1, Dvl2, and Dvl3 are expressed at varying levels in mouse totipotent F9 embryonal teratocarcinoma cells. The expression of each endogenous Dvl isoform, as defined by knock-down with siRNA, was obligate for Wnt5a to activate NF-AT-sensitive transcription. Elements upstream of effectors, *e.g*., cGMP phosphodiesterase and Ca^2+^-mobilization, were blocked by knock-down of any one of the Dvls; thus, with respect to Wnt5a activation of NF-AT Dvls are not redundant. Among the three Dvl isoforms, the C-terminal sequence of Dvl3 is the most divergent. Deletion of region of Dvl3 abolishes Wnt5a-stimulated signaling. Alanine (Ala)-substitution of histidine (His) single amino acid repeats at 637,638 and/or 647,648 in Dvl3, like C-terminal deletion, abolishes Wnt 5a signal propagation. Phenylalanine (Phe)-substitution of the same His-repeats in Dvl3 mimics Wnt5a stimulated NF-AT-sensitive transcription.

**Conclusions:**

The C-terminal third of Dvl3 and His single amino acid repeats 637,638 and 647,648 (which are unique to and conserved in Dvl3) are essential for Wnt5a activation of the non-canonical pathway, but not the Wnt3a activation of the canonical pathway.

## Background

Wnt signaling is essential for normal embryonic patterning, development, cellular proliferation and homeostasis [1-4]. Wnt ligands initiate intracellular signaling pathways by binding to the G-protein-coupled receptors (GPCR), Frizzleds (Fzs) [5-9]. The Wnt-sensitive pathways include the canonical (Wnt/β-catenin) and the non-canonical (planar cell polarity and Wnt/Ca^2+^) pathways [10-15]. For the canonical pathway, absent Wnt, cellular β-catenin is subjected to proteasome mediated degradation by the destruction complex that includes, among other proteins, Axin and the product of the *adenomatous polyposis coli *gene, which facilitate the phosphorylation of β-catenin by the Ser/Thr protein kinase glycogen synthase kinase 3β[16-20]. This phosphorylation that occurs in the absence of Wnt3a fosters ubiquitination and degradation of β-catenin [21-23]. In the presence of Wnt, activation of Frizzled receptor leads to inhibition of glycogen synthase kinase 3β and stabilization of β-catenin [24-26], both of which are Dvl-dependent[17,27]. Nuclear accumulation of β-catenin yields activation of lymphoid-enhancer factor/T-cell factor (Lef/Tcf)-sensitive transcription of developmentally-related genes [5,28]. Post-transcriptional regulation of β-catenin mRNA also plays a role in Wnt-stimulated regulation of β-catenin [29].

Wnt5a, operating via Frizzled-2, leads to activation of cyclic GMP phosphodiesterase (cGMP PDE) [30-32], a decline in intracellular cyclic GMP, and sharp transient increase of intracellular Ca^2 ^[32-35]. This Wnt5a-stimulated mobilization of Ca^2+ ^activates the protein phosphatase, calcineurin, which dephosphorylates a transcription factor, the Nuclear Factor of Activated T cells (NF-AT) and stimulates translocation of NF-AT from cytoplasm to nuclei [36] where transcription of NF-AT-sensitive genes is activated [34,36]. That *WNT5A *is a cancer-associated gene implicated in the invasion and metastasis of several human cancers, including colorectal [37], breast and pancreas [38], heightens the need to more fully understand the function and dysfunction of the Wnt non-canonical pathway.

Mammalian cells express three isoforms of the phosphoprotein Dishevelled (Dvl) [39-42], a scaffold protein harboring three well-known highly-conserved domains, i.e., DIX, PDZ, and DEP [43,44]. For example, Wnt5a has been shown to induce Dvl phosphorylation via casein kinase 1, altering the distribution of Dvl [45]. The DIX domain is essential for Dvl-Dvl or Dvl-Axin dimerization [17]. DIX and PDZ domains are necessary for Wnt canonical signaling. Deletion of either DIX or PDZ domain blocks Wnt canonical pathway [17,46,47]. The DEP domain is essential for the PCP signaling, in tandem with Daam [44,48,49]. C-terminal to the DEP domain is the region of greatest dissimilarity among Dvl1, Dvl2, and Dvl3. Three isoforms of Dvl appear to function cooperatively as well as uniquely with respect to mediation of Wnt3a-stimulated canonical signaling [50]. Dishevelled has been implicated as mediating Wnt5a-stimulated activation of PKC [51], but what role mammalian Dvls play in Wnt5a-stimulated regulation of the overall pathway to activation of NF-AT-sensitive gene transcription is unknown. We sought to answer the fundamental question of whether or not Dvl isoforms are functionally redundant with respect to the regulation of the non-canonical pathway by Wnt. Furthermore, we sought to interrogate the C-terminal region of mammalian Dvls in particular, since this is the region of these molecules that diverge most at the level of sequence. Finally, we sought to investigate the role of two single His-repeats found in the distal region of the C-terminus of Dvl3. These single His-repeats are unique to only a single member of the Dvl family, Dvl3. We make use of totitpotent mouse F9 teratocarcinoma cells, a useful model of early development [52,53] and differentiation [54]; we interrogated each question, establishing that the non-canonical pathway is most sensitive to suppression of Dvl3 (although all three Dvls are necessary for normal signaling) and that conserved His-repeats unique to C-terminus of Dvl3 are obligate for non-canonical signaling of Wnt5a to the NF-AT-dependent transcriptional response.

## Results

### Dishevelleds mediate Wnt-stimulated NF-AT activation

**Figure 1:**
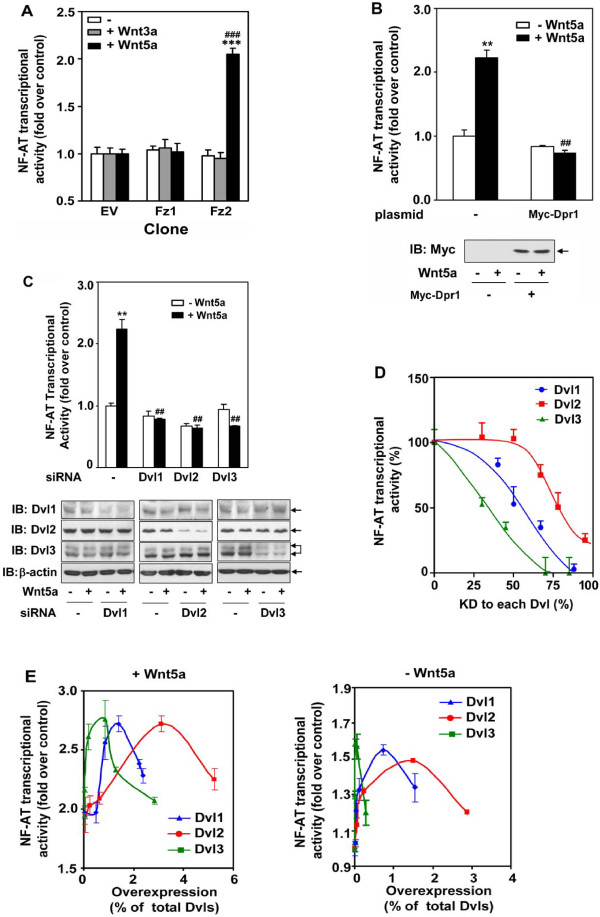
**Dvl1, Dvl2, and Dvl3 expression are each required for Wnt5a/Fz2/NF-AT signaling**. ***A***, mouse F9 stable clones expressing rat Frizzled1 (Fz1), rat Frizzled2 (Fz2) or empty vector (EV) [30] were treated with vehicle (-), Wnt3a or Wnt5a for 6 hours and the activity of NF-AT-dependent luciferase was measured. ***p < 0.001, compared with corresponding vehicle-treated group; ###, *p *< 0,001, compared with Wnt5a-treated "EV" group. ***B***, Myc-Dapper1 (Myc-Dpr) was expressed (assayed by immunoblotting, bottom panel) in F9 clones expressing Fz2. The activity of NF-AT reporter in response to Wnt5a was determined as described. ***C***, Suppression of Dvl1, Dvl2, or Dvl3 using siRNA targeting a single Dvl isoform was conducted in F9 clones expressing Fz2. The activation of NF-AT-sensitive transcription in the presence (+) or absence (-) of Wnt5a was assayed by using a luciferase reporter. **, *p *≤ 0.01, compared with basal (-Wnt5a) in control group; ##, *p *≤ 0,01, compared with Wnt5a-treated (+Wnt5a) control group. Suppression of Dvl1, 2 or 3 by using siRNA was examined by immunoblotting. The blots of actin were shown as loading controls. ***D***, F9 cells expressing Fz2 were treated with variable amounts of siRNAs targeting a single Dvl isoform. The activation of the NF-AT-dependent luciferase in response to Wnt5a was measured. The data are plotted as "% of maximal" of the Wnt5a-sensitive luciferase response *versus *the extent of knock-down (KD) in individual Dvl isoforms produced by treatment of increasing amounts of siRNA. The level of Knock-down for each Dvl was verified by immunoblotting (data not shown). ***E***, Increased amounts of Dvl were expressed in F9 clones expressing Fz2. The activation of NF-AT-sensitive transcription in the presence (left panel) or absence (right panel) of Wnt5a was assayed by using a luciferase reporter. The luciferase activities were plotted *versus *the amount of immunoreactive staining for the HA-tag quantified in the blots (data not shown).

Totipotent mouse embryonal carcinoma F9 cells transfected to express rat Frizzled-2 (Fz2) provided an optimal system for the biochemical analysis of Wnt5a/Fz2 signaling [52,53]. Upon treatment with Wnt5a, F9 cells differentiate into primitive endoderm, characteristic of early mouse development [8]. Stimulating F9 cells with Wnt5a provoked activation of NF-AT-sensitive transcription (Figure 1A). This marked activation of NF-AT-sensitive gene transcription was provoked in cells expressing Frizzled-2, but not in those expressing either Frizzled-1 (Fz1) or empty vector (EV). Mouse F9 cells express all three isoforms of Dishevelled [50], i.e., Dvl1, Dvl2, and Dvl3 (Figure 1B). Dapper1 acts as a negative modulator of the canonical Wnt3a/β-catenin signaling, antagonizing Dvl function [55], so our first test of the role of Dvl in the non-canonical pathway was expression by use of Dapper1. F9 cells were transiently transfected with an expression vector harboring a Myc-tagged version of mouse Dapper1 (verified by immunoblotting, Figure 1B). The expression of Dapper1 clearly abolished the ability of Wnt5a to activate NF-AT-sensitive transcription. Thus, we concluded that Dishevelleds play an essential role in mediating Wnt5a-stimulated non-canonical signaling to the level of NF-AT-dependent transcription.

### Dishevelleds-1, -2, and -3 are each essential for Wnt5a activation of the non-canonical pathway

The individual roles of each mammalian Dvl isoform in Wnt5a-stimulated activation of NF-AT-sensitive transcription were interrogated by treating cells with Dvl-isoform-specific siRNAs. To preclude misleading possible "off-target" effects associated with siRNA treatment, we routinely employ two or more distinct siRNA targeting sequences for every target, in this case for each of the three mammalian Dvls. The treatment of cell with siRNA individually suppressed Dvl1, Dvl2, and Dvl3 (Figure 1C); each siRNA suppressed expression of only its targeted Dvl isoform. Treatment with "control", scrambled siRNAs designed by the commercial supplier were without effect (data not shown). The relative levels of expression of each Dvl has been reported in mouse F9, mouse P19, and human HEK cells; the rank order of expression is Dvl2 > > > Dvl3 > Dvl1 [50]. Expression of each Dvl isoform was knocked-down by > 80%.

Knock-down (KD) of any one of the mammalian Dvl isoforms abolishes the ability of Wnt5a to regulate the overall pathway to the level of NF-AT-dependent transcriptional activation (Figure 1C). As noted, KD provoked by an additional set of siRNAs targeting the same isoforms yielded identical results (additional file 1). Thus, KD of either Dvl1 alone, or of Dvl2, or of Dvl3 all abolished activation of NF-AT-sensitive transcription in response to Wnt5a. We conclude that Dvl isoforms do not display frank "redundancy". Knock-down of either the most abundant Dvl isoform (*i.e*., Dvl2, constituting > 90% of Dvl pool) or Dvl1 (< 5% of the Dvl pool) or Dvl3 (< 3% of the pool), each blocks Wnt5a activation of the non-canonical pathway.

### Non-canonical signaling: differential sensitivity to loss of individual Dvls

Differential sensitivity of the canonical Wnt/β-catenin/Lef-Tcf-dependent transcriptional pathway to loss of individual mammalian Dvl1, Dvl2, and Dvl3 has been shown [50]. Since KD of any one of the individual Dvl isoforms succeeded in abolishing the overall Wnt5a-stimulation of NF-AT-sensitive transcriptional, we probed if this non-canonical signaling displays differential sensitivity to the level of expression of individual Dvls. Cells were treated with sufficient amounts of siRNAs targeting the expression of an individual Dvl isoform to achieve varied levels of expression from ~80% to ~20%, as established by immunoblotting (data not shown). Curves plotting Dvl expression versus NF-AT-sensitive transcription are displayed (Figure 1D) and were remarkable in several respects. KD of ~25% of Dvl3 provoked about a 50% reduction in Wnt5a-stimulated non-canonical signaling. On the other hand, KD of Dvl2, which constitutes > 90% of the Dvl pool, had to reach > 65% before the overall Wnt5a-sensitive non-canonical pathway was impacted. The effects of the stepwise KD of Dvl1 displayed a curve intermediate between those for KD of Dvl3 and of Dvl2 (Figure 1D). Thus, the Wnt5a-stimulated non-canonical pathway appears to display the same rank order for sensitivity to graded KD of individual mammalian Dvl isoforms (Figure 1D), as was noted for Wnt3a-stimulation of the canonical β-catenin pathway[50], i.e., Dvl3 > Dvl1 > > Dvl2.

### Non-canonical signaling: differential sensitivity to overexpression of Dvls

Transient expression of Dishevelleds has been shown to activate the Wnt canonical pathway in the absence of Wnt3a [50]. We sought to interrogate if overexpression of individual Dvl isoforms might impact the ability of Wnt5a-stimulated pathway to operate in either the presence or absence of Wnt5a. Overexpression of Dvl1, or Dvl2, or Dvl3 was found to activate the non-canonical pathway (Figure 1E). The effect of overexpression of each Dvl isoform on the response was bell-shaped. Generally, activation of the non-canonical pathway increased with increasing expression of any one of the mammalian Dvl isoforms. When the Wnt5a-stimulated response was the output, the effects of overexpression of any one Dvl peaked at ~2.75-fold over basal. There above, increased expression of each isoform uniformly attenuated the response. The rank order (from most to least effective) for the ability of a Dvl to potentiate the Wnt5a-stimulated NF-AT-sensitive transcriptional response was Dvl3 > Dvl1 > Dvl2. This same rank order was displayed for the response to increased expression of Dvl isoform alone, in the absence of Wnt (- Wnt5a). Dvl3 appears to be most intimately involved in the Wnt5a-stimulated non-canonical response. The NF-AT response is most sensitive to decreases in Dvl3 that attenuate the non-canonical pathway as well as to overexpression of Dvl3 that potentiate the response to Wnt5a.

### Dvls impact the Wnt non-canonical pathway upstream of cGMP and intracellular Ca^2+^mobilization

**Figure 2:**
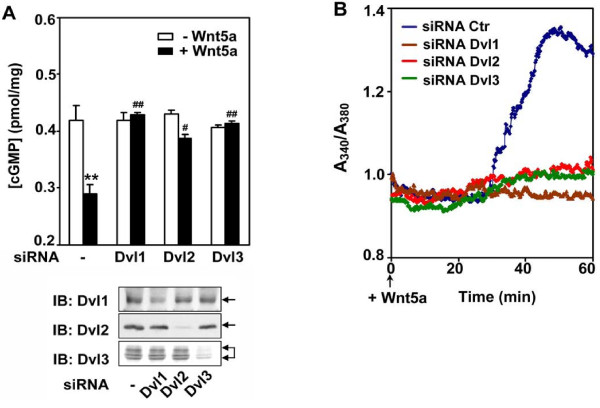
**Dvl3 is requisite in Wnt5a/Fz2/cGMP/Ca^2+^signaling**. ***A*, **Fz2 expressing cells were treated with control siRNA (-) or siRNA targeting individual Dvl isoforms. Cells were treated with vehicle (-Wnt5a), or Wnt5a (+Wnt5a) for 45 min and cellular cGMP was assayed (left panel). Suppression of Dvl1, 2 or 3 by using siRNA was examined by immunoblotting (right panel). ***B***, cells were treated for 48 hr with either control siRNA (Ctr) or siRNA targeting individual Dvl isoforms. After loaded with Fura2, cells were challenged by Wnt5a (+Wnt5a). A representative graph of Ca^2+ ^transient presented as A_340_/A_380 _for cells treated with different siRNA presented. Each line depicts the mean values obtained from 20 cells selected randomly.

We sought to discern the position at which mammalian Dvls acted in the Wnt5a-stimulated non-canonical pathway. The overall non-canonical pathway from Wnt5a treatment to activation of NF-AT-dependent transcription was blocked by Dapper1 as well as by KD of Dvl1, Dvl2, or Dvl3 (Figure 1). The ability of KD of individual Dvl isoforms to influence signals downstream of heterotrimeric G-proteins [53] was interrogated at the level of the G-protein effector, cGMP phosphodiesterase (Figure 2A). In cells expressing endogenous levels of each mammalian Dvl, Wnt5a activated cyclic GMP phosphodiesterase provokes a sharp decline in intracellular cyclic GMP (cGMP). The siRNA-induced KD of each Dvl individually abolished the ability of Wnt5a to activate cGMP phosphodiesterase, as read-out by the degradation of intracellular cGMP (Figure 2A). The response to Wnt5a in Dvl isoform-deficient cells was probed downstream, at the level of Ca^2+ ^mobilization [34,56]. Ca^2+ ^mobilization was activated by Wnt5a in the control mouse F9 cells (Figure 2B). The KD of individual Dvls abolished the ability of Wnt5a to provoke the Ca^2+^-mobilization. Thus, Dvl1, Dvl2, and Dvl3 appear to be modulating the Wnt5a non-canonical signaling upstream of cGMP phosphodiesterase, a well known G-protein effector, mediating Wnt5a effects.

### Co-localization of Dvls

**Figure 3:**
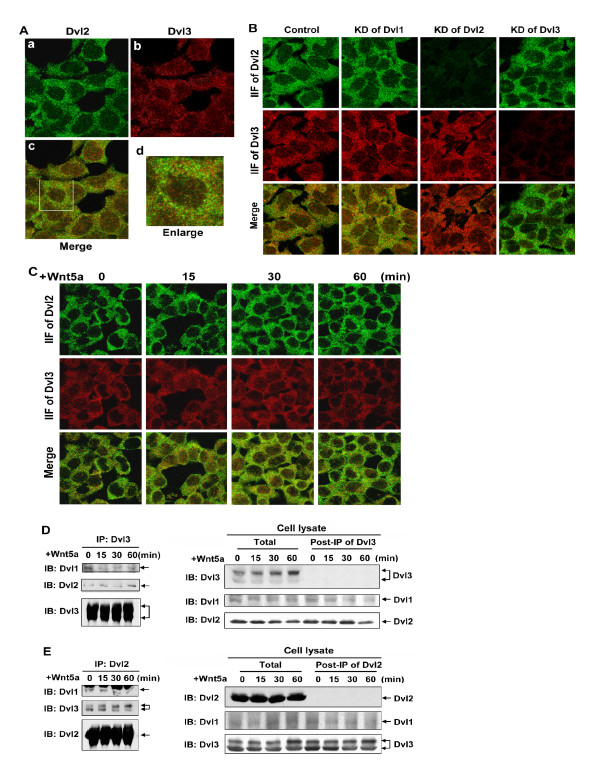
**Dvl2 and Dvl3 form punctate complexes**. ***A***, Fz2 expressing cells were cultured on a cover-glass in a MatTak culture dish. Cells were fixed and Dvl2 and Dvl3 were doubly stained by using antibodies against Dvl2 or Dvl3. ***B***, cells were treated with siRNA targeting individual Dvl isoforms for 2 days and cultured on a cover-glass in a MatTak culture dish. Cells were fixed and Dvl2 and Dvl3 were doubly stained by using antibodies against Dvl2 or Dvl3. ***C***, cells were treated with Wnt5a for indicated time periods. Cells were fixed and cellular Dvl2 and Dvl3 were doubly stained by using specific antibodies against Dvl2 or Dvl3, respectively. ***D ***and ***E***, cells were treated with Wnt5a for indicated time periods. Immunoprecipitation-based "pull-downs" from whole-cell lysates were performed by using specific antibodies that either recognizes only Dvl3 (panel ***D***), or recognizes only Dvl2 (panel ***E***). The Dvl-based complexes isolated in the pull-downs ("IP:Dvl3" and "IP:Dvl2"; left panels in ***D ***and ***E***, respectively), whole-cell lysates ("Total"; right panel), as well as supernatant fraction following the immune precipitation ("post-IP of Dvl3" and "post-IP of Dvl2"; right panels in ***D ***and ***E***, respectively) were subjected to SDS-PAGE and the resolved proteins transferred to nitrocellulose blots. The blots were stained with antibodies specific for each Dvl isoform. A set of representative images of immunoblotting are shown.

Dvls share high homology in primary sequence, *i.e*., the overall identity of Dvl1 to Dvl2 is 63.6%; the overall identity of Dvl3 to Dvl2 is 71.5%. Each Dvl also displays a common set of motifs, including DIX, PDZ and DEP domains. The localization of Dvl2 and Dvl3 was interrogated by immunohistochemical methods using AlexaFluor488-conjugated secondary antibody to stain Dvl2 (green) and AlexaFluor595-conjugated secondary antibody to stain Dvl3 (red, Figure 3A). Labeling patterns for both Dvl2 (panel *a*) and Dvl3 (panel *b*) are punctate, localized throughout the cytoplasm (Figure 3A). These patterns indeed are similar to those reported earlier in studies of Dvl2 alone [40,57]. By merging images, we established that the bulk of either Dvl2 or Dvl3 is not co-localized (panels *c, d*). For the highly abundant Dvl2, co-localization of < 20% with Dvl3 would not have been unexpected. The presence of Dvl3 (the least abundant mammalian Dvl in these cells) not co-localized with the ~18-fold more abundant Dvl2 in these cells was not anticipated and was therefore of great interest. We next extended these efforts at interrogating the extent of Dvl co-localization (of Dvl2 and Dvl3) to cells made deficient of a single Dvl isoform (Figure 3B). In cells made deficient of any one Dvl, co-localization of Dv2 with Dvl3 was apparent, but again only partial (see panel *"merge"*). Remarkably, there was no major change in punctate patterns observed immunohistochemically in the distribution/localization of Dvl2 or Dvl3 in cells made deficient of another Dvl (Dvl1) by siRNA treatment. The KD of Dvl1 had no effect on the expression, localization, and co-localization of Dvl2 and Dvl3. Patterns of either Dvl2 or Dvl3 as well as their co-localization (*"merge" *panels) were similarly seemingly unaffected by Wnt5a (Figure 3C).

As a second approach with which to probe co-localization of Dvl isoforms, immuneprecipitation (IP) of Dvls were employed in pull-downs from lysates prepared from untreated and Wnt5a-treated cells. The analysis was conducted with IPs of Dvl2 and of Dvl3 (anti-Dvl1 antibodies were unable to affect a quantitative pull-down of their targets from cell lysates). IPs and supernatants of samples of cell lysates probed following the immuneprecipitation were subjected to sodium dodecyl sulfate polyacrylamide gel electrophoresis (SDS-PAGE) and then immunoblotting (IB). These biochemical results confirm the results obtained from immunohistochemical co-localization studies, *i.e*. Dvl2 and Dvl3 clearly do associate (left panels of Figure 3D, E). However, less than 10% of each Dvl is found in association with the other Dvl under these conditions of immunoprecipitation. Treating the cells with Wnt5a yielded no detectable differences of Dvl content in the analysis of fractions both pre- and post-IP (right panel of Figure 3D, E). Thus, such partial co-localization of Dvl isoforms would not seem a likely explanation by which to rationalize the essential character of each Dvl isoform regarding its function in Wnt5a-stimulated non-canonical signaling.

### Non-canonical signaling in Dvl-deficient cells

**Figure 4:**
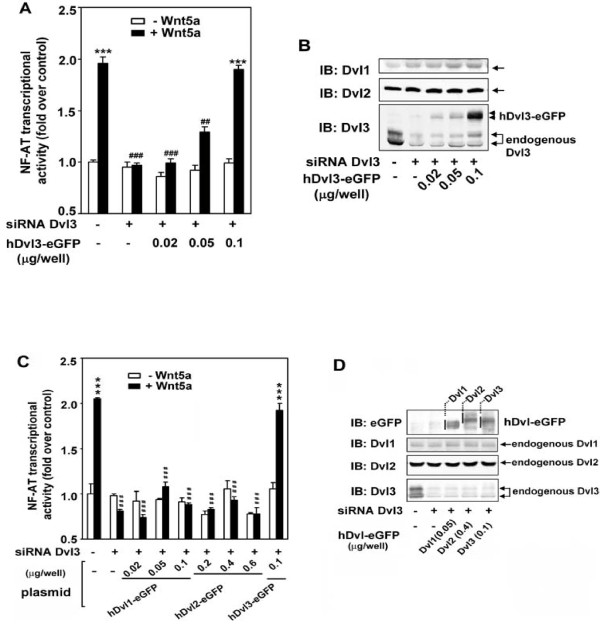
**Expression of hDvl3, but neither hDvl1 nor hDvl2, rescues Wnt5a-stimulated NF-AT-sensitive transcriptional activation in Dvl3-depleted cells**. F9 cells stably expressing Fz2 were treated with siRNA targeting Dvl3. Dvl3-depleted cells were transfected with various amounts of expression plasmid harboring hDvl3-eGFP (***A ***and ***B***), or hDvl1-eGFP or hDvl2-eGFP (***C ***and ***D***). The Wnt5a-sensitive transcriptional activation was performed by the assay of NF-AT-dependent luciferase reporter (***A ***and ***C***). ***, *p *≤ 0.001, compared with basal (-Wnt5a) in control (-siRNA, -plasmid) group; ^##^, *p *≤ 0.01, ^###^*p *≤ 0.001, compared with (+Wnt5a) in control group. The cell lysates from the same batch used in reporter assay were subjected to SDS-PAGE and the resolved proteins transferred to nitrocellulose blots. The blots were stained with antibodies indicated (***B ***and ***D***). A set of representative images of immunoblotting are shown.

The degree of homology that exists among mammalian Dvl1-3 fostered our study of whether or not overexpression of one isoform might possibly "rescue" the absence of Wnt5a-stumulated NF-AT-dependent transcription in the Dvl3-deficient cells. Is it possible to overcome the absence of Dvl3 that constitutes < 5% of pool through overexpression of the remaining two isoforms? Cells were made Dvl3-difficient by siRNA treatment. Dvl3-deficiency abolished Wnt5a-stimulated NF-AT-sensitive transcription (Figure 4A, C), as shown before (Figure 1). Loss of endogenous Dvl3 expression showed no detectable influence on the expression level of the Dv2. A slight increase in Dvl1 expression was observed, although non-canonical signaling remained abolished. We transiently transfected the Dvl3-deficient F9 cells with an expression vector harboring eGFP-tagged human Dvl3 (hDvl-eGFP). Expression of hDvl3 at the level that was compatible with that of endogenous Dvl3 expressed in non-siRNA treated cells fully rescues the function of the non-canonical signaling (Figure 4A, B). Using this same Dvl3-depletion and rescue strategy, we ascertained if expression of either Dvl1 or Dvl2 also would rescue NF-AT-dependent transcriptional activation in Dvl3-deficient cells (Figure 4C, D). Neither Dvl1 nor Dvl2 was able to rescue non-canonical signaling in response to Wnt5a, although expressed to levels equal to or greater than those achieved by hDvl3-eGFP, which fully rescued the NF-AT-dependent transcription in response to Wnt5a. Only expression of hDvl3 rescues non-canonical signaling in response to Wnt5a in cells made deficient in the endogenous Dvl3, i.e., Dvl3 function is not redundant with either Dvl2 or Dvl1.

### DIX domain and distal C-terminal region of Dvl3 is essential for Wnt5a-stimulated non-canonical signaling

**Figure 5:**
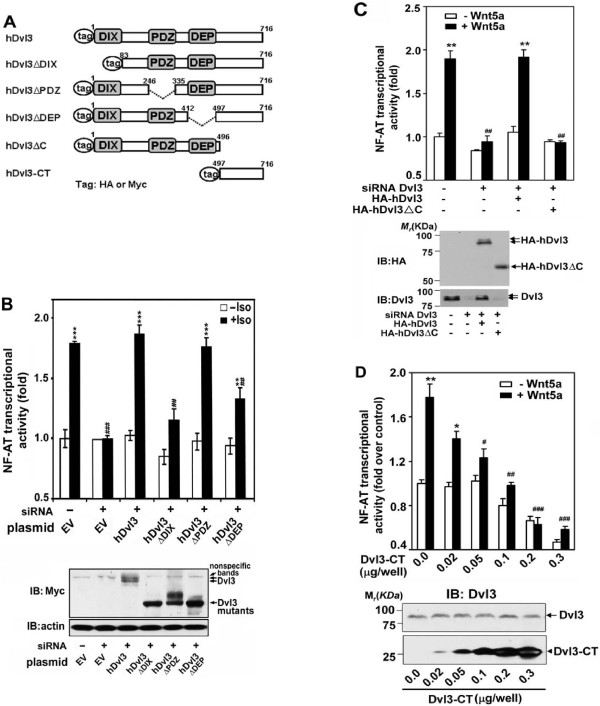
**The DIX domain and C-terminal region of Dvl3 are obligate for non-canonical signaling**. ***A***, a schematic of deletion mutants of human Dvl3. ***B, ***F9 cells expressing β_2_AR/Fz2 were made deficient in Dvl3 by treating cells with siRNA targeting Dvl3. Myc-tagged hDvl3 or mutants (i.e. hDvl3ΔDIX, hDvl3ΔPDZ, hDvl3ΔDEP) were then expressed in these cells and NF-AT-dependent reporter activity induced by activation of the pathway (+Iso, 10 μM) was measured and displayed as "fold-activation", setting reporter activity in control cells (-siRNA, EV) as "1" (upper panel). **, *p *≤ 0.01, ***, *p *≤ 0.001 compared with basal (-Iso) in control (-siRNA, EV) group; ^## ^*p*, ≤ 0.01, ^###^*p *≤ 0.001, compared with stimulation (+Iso) in control group. Expression of hDvl3 or mutants was analyzed by immunoblotting (bottom panel). The blots of β-actin were shown as loading controls. ***C***, F9 cells expressing Fz2 were treated with or without siRNA targeting Dvl3. Twenty four hours after siRNA treatment, cells were transfected with expression vector harboring either HA-hDvl3 or HA-hDvl3ΔC. Wnt5a-stimulated NF-AT reporter activity was measured (upper panel). **, *p *≤ 0.01, compared with basal (-Wnt5a) in control (-siRNA) group; ^##^, *p *≤ 0.01, compared with (+Wnt5a) in control group. Cell lysates were subjected to SDS-PAGE followed by immunoblotting (IB). Results from enhanced chemical luminescence (ECL) of the exposed blots are displayed (bottom panel). ***D***, Various amounts of HA-tagged hDvl3-CT were expressed in F9 cells expressing Fz2. The activity of NF-AT luciferase reporter in response to Wnt5a was assayed (upper panel). *, *p *≤ 0.05, **, *p *≤ 0.01, compared with basal (-Wnt5a) in control group; ^#^, *p *≤ 0.05, ^##^, *p *≤ 0.01, ^###^*p *≤ 0.001, compared with Wnt5a-treated control group. Expressions of Dvl3-CT as well as endogenous Dvl3 in cell lysates were analyzed by immunoblotting (bottom panel).

Using the same "rescue" strategy with Dvl3-deificient cells, we tested the ability of Dvl3 deletion mutants to mediate the Wnt5a-stimulated non-canonical signaling. Sequence homology among the three mammalian Dvls is greatest in the N-terminal region extending to approximately reside 500, and includes three well-known domains, i.e., DIX, PDZ, and DEP [43,44]. Dvl sequence C-terminal to the DEP domain displays the least homology among Dvls. We constructed mutants of Dvl3 with deletions of the DIX, the PDZ, the DEP domains or of the C-terminal third of the molecule (Figure 5A). Since these various domains participate in other Wnt signaling pathways, we employed activation of the non-canonical pathway via the β_2_-adrenergic receptor/rat Frizzled2 chimera (Figure 5B) [8,30,34]. We tested if the deletion mutants could "rescue" the activated non-canonical pathway to the level of NF-AT-dependent transcription in Dvl3-deficient F9 cells. Expression of the wild-type human Dvl3 fully rescued the non-canonical signaling pathway. The loss of the PDZ domain was without effect on the signaling, while the deletion of the DIX domain nearly abolished the ability of the mutant to rescue the response in Dvl3-deficient cells. Expression of the mutant Dvl3 lacking the DEP domain only partially rescued the non-canonical signaling. Human Dvl3 lacking the C-terminal 497-716 region (hDvl3ΔC) abolished the ability to rescue the non-canonical signaling pathway in Dvl3-deficient cells (Figure 5C). Expression of full-length hDvl3, but not the hDvl3ΔDIX nor the C-terminal truncated mutant (hDvl3ΔC), was able to fully rescue the Wnt5a-stimulated NF-AT response. Thus, although the hDvl3ΔC mutant harbors DIX, PDZ, and DEP domains as well as 70% of the wild-type sequence, it cannot rescue non-canonical signaling to the level of NF-AT-dependent transcription in the mDvl3-deficient cells.

### Expression of hDvl3-CT blocks the NF-AT activation response to Wnt5a

As a second strategy with which to interrogate the functional role of the C-terminal domain of Dvl3 in Wnt5a-stimulated non-canonical signaling, we probed if the same C-terminal region, expressed as a peptide in F9 cells, might display some effect on the signaling pathway. We created an expression vector harboring the hDvl3 C-terminal region (amino acid 497-716, hDvl3-CT), expressed the peptide in F9 cells, and probed Wnt5a-stimulated NF-AT activation (Figure 5D). Expression of hDvl3-CT fragment, even at the lowest amount of input DNA tested (i.e., 0.02 μg/well), actively suppressed Wnt5a-stimulated NF-AT-sensitive transcription. Increasing the expression of the hDvl3-CT fragment provoked even greater attenuation of the Wnt5a-stimulated NF-AT response. At 0.1 μg input DNA/well, the hDvl3-CT fragment not only reduced the Wnt5a-stimulated response to basal levels, but also demonstrated the capacity to suppress the ambient NF-AT-dependent transcription, measured in the absence of Wnt5a. The ability of the expression of the Dvl3-CT fragment to suppress Wnt5a-activation of the non-canonical signaling suggests a dominant-interfering character. Thus the C-terminus of Dvl3 is essential for its role in mediating the Wnt5a stimulation of NF-AT-dependent transcription.

### Deletion of His single amino acid repeats (residues 630-650) abolishes Dvl3 function in Wnt/NF-AT non- canonical signaling

**Figure 6:**
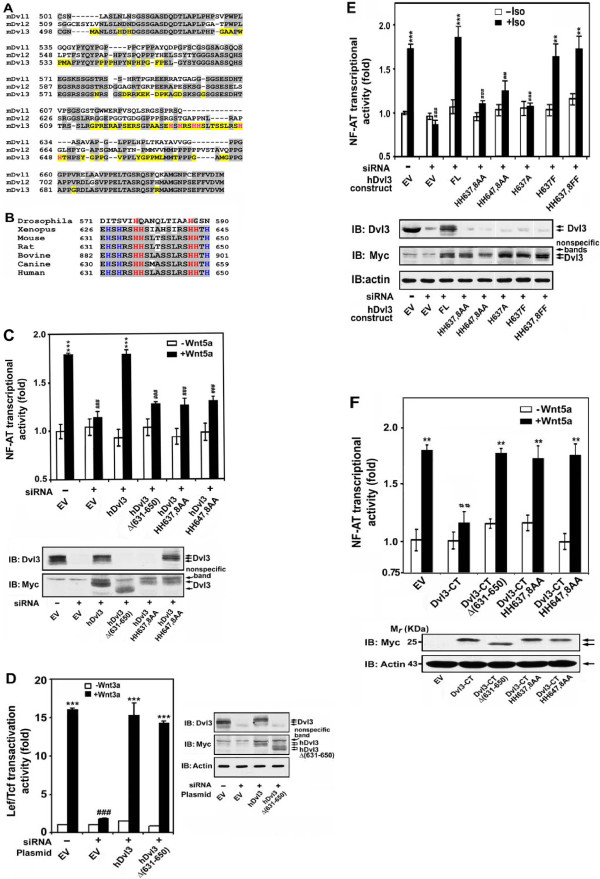
**Single His-repeats at 637,638 and 647,648 are essential for Dvl3 function in Wnt5a/cGMP/Ca^2+ ^non-canonical signaling to NF-AT-dependent transcription**. ***A***, sequence alignment of C-terminus of mouse Dvl1, 2, and 3 is displayed. Sequences that are common to at least two mDvls are shaded in gray. Unique sequences of mDvl3 are highlighted in yellow. His residues located within amino acid 631-650 of mDvl3 are displayed in red. ***B***, Sequence alignment of mouse Dvl3 (631-650) with corresponding region of Dvl from fly Dsh, frog Dvl3 and mammalian Dvl3 is displayed. Common sequences are shaded in gray. HH637, 638 and HH647, 678 of mouse Dvl3 and corresponding single His-repeats of Dvl3 in other species are displayed in red. Other His residues in the region are displayed in blue. ***C***, F9 cells stably expressing Fz2 were treated with siRNA targeting Dvl3 (siRNA). Dvl3-depleted cells were transfected with an empty vector (EV) or expression vector harboring either Myc-hDvl3, or one of hDvl3 mutants as indicated. Activity of NF-AT-reporter was measured in response to Wnt5a (upper panel). ***, *p *≤ 0.001, compared with basal (-Wnt5a) in control (-siRNA and EV) group; ^##^, *p *≤ 0.01, compared with (+Wnt5a) in control group. Expressions of Myc-hDvl3 or mutants were analyzed by immunoblotting by using anti-Dvl3 and anti-Myc antibodies (bottom panel). ***D***, F9 cells stably expressing Fz1 were treated with siRNA targeting Dvl3 (siRNA). Rescue experiments were conducted by expression of either Myc-hDvl3, or Myc-hDvl3Δ(631-650) in Dvl3-depleted cells, followed by challenging cells with or without Wnt3a. Activity of Lef/Tcf-dependent transcription was measured by using a luciferase reporter (left panel). ***, *p *≤ 0.001, compared with basal (-Wnt3a) in control (-siRNA and EV) group; ^###^, *p *≤ 0.001, compared with (+Wnt3a) in control group. Expressions of Myc-hDvl3 or Myc-hDvl3Δ(631-650) were analyzed by immunoblotting (right panel). ***E***, F9 cells expressing β_2_AR/Fz2 were treated with siRNA targeting Dvl3 (siRNA). Rescue experiments were conducted by expression of Myc-hDvl3 (FL), or one of Myc-hDvl3 mutants indicated, followed by activating the non-canonical pathway by treating cells with or without isoproterenol (Iso). Activity of NF-AT-reporter was measured and analyzed (upper panel). **, *p *≤ 0.01, ***, *p *≤ 0.001, compared with basal (-Iso) in control (-siRNA and EV) group; ^##^, *p *≤ 0.01, ^###^, *p *≤ 0.001, compared with (+Iso) in control group. The expression of each hDvl3 variant was evaluated by immunoblotting by using anti-Dvl3 and anti-Myc antibodies. The same blots were stained with anti-β-actin antibody (IB: actin) to establish loading equivalence. ***F***, Dvl3-CT, Dvl3-CTΔ(631-650), Dvl3-CT HH637,8AA, or Dvl3-CT HH647,8AA was expressed in F9 clones stably expressing Fz2. The activity of NF-AT-dependent luciferase reporter was measured following stimulation of Wnt5a (upper panel). **p ≤ 0.01, compared with basal (-Wnt5a) in control (EV) group; ^##^, ≤ 0.01, compared with (+Wnt5a) in control group. Expression of Dvl3-CT or mutants at a similar level was shown by immunoblotting (bottom panel). The blots of β-actin are shown as loading controls.

In an effort to further uncover the nature of the Dvl3 C-terminal involved in NF-AT activation in response to Wnt5a, we compared the sequences of the C-terminal region of all three mammalian Dvls (Figure 6A). The three mammalian Dvls display considerable homology within approximately 2/3rds of the C-terminal region. Of particular interest to us was the uniqueness of single amino acid His-His repeats at two positions (His 637, 638 and His 647, 648) of the C-terminus of Dvl3 among mammalian Dvl3. These two His single amino acid repeats in Dvl3 are highly conserved from *Xenopus *to human (Figure 6B). Only at the level of the fly (*Drosophila*, which displays only the single *Dishevelled*) is the His repeat apparently absent. Employing the KD-rescue strategy, we showed that the integrity of the C-terminus (residue 631-650 containing His tandem repeats) was essential for Dvl3 to mediate Wnt5a-stimulated non-canonical signaling to NF-AT-dependent transcription (Figure 5C). We extended this observation further and tested if this same region, unique to Dvl3 and essential to non-canonical signaling, functioned similarly in the Wnt3a-stimulated canonical β-catenin and Lef/Tcf-dependent transcriptional response. The Dvl3 (Δ 631-650) mutant was expressed in Dvl3-deficient cells, and the Lef/Tcf-dependent transcription sensitive to Wnt3a analyzed (Figure 6D). The (Δ 631-650) mutant, not capable of rescuing the non-canonical pathway, fully rescued the Wnt3a-stimulated canonical pathway. Thus not only do we show that Dvl3 has a character that can be differentiated from Dvl1 and Dvl2, but also that the C-terminal region unique to Dvl3 is essential for non-canonical signaling but dispensable for canonical signaling.

### Mutagenesis of His tandem repeats either 637,638 or His 647,648 abolishes Dvl3 function

We sought then to focus on specific mutation of the single His-repeats conserved uniquely in the distal region of the Dvl3 (Figure 6A, B). Alanine-substitution of either His-repeat (637,638 or 647,648) abolished the ability of the Dvl3 mutants to rescue the activation of the non-canonical pathway (Figure 6C, E). Alanine-substitution of either His residue within the single His-repeat (637,638) likewise abolished the ability of the Dvl3 mutant to function in the non-canonical signaling to NF-AT-dependent transcription (Figure 6E, Table 1). We exhaustively interrogated His residues in the target region of 631-650 of Dvl3 sequence by alanine-substitution (Figure 6E and Table 1). Whereas mutagenesis of the single His-repeats essentially abolished Dvl3 function in the non-canonical pathway, alanine substitution of His residues elsewhere either only attenuated Dvl3 function (i.e. His 632, His 650), or had no significant functional outcome at all (His 634). The functional character of the alanine mutant at 637 was restored to normal if the mutation was reversed or the alanine was substituted with the more bulky phenylalanine substitution (Figure 6E).

**Table 1:** Comparison of His mutants of hDvl3 in the ability of rescuing Wnt non-canonical signaling.

hDvl3 mutants	Rescue (%)
H637A	9 ± 4
H638A	9 ± 7
H647A	36 ± 8
H648A	33 ± 8
H632A	72 ± 9
H634A	91 ± 1
H650A	61 ± 8

F9 cells expressing β_2_AR/Fz2 were treated with siRNA targeting Dvl3 (siRNA). Rescue experiments were conducted by expression of hDvl3, or hDvl3 mutants containing His to Ala point mutation as indicated. The non-canonical pathway was activated by treating cells with or without isoproterenol (10 μM). Activity of NF-AT-reporter was measured and analyzed, setting the full rescue by hDvl3 as 100%.

Finally, we further probed the observations obtained from alanine-substitution mutagenesis that reveals the single His-repeats at 637,638 and at 647,648 to be essential for Dvl3 to function in the Wnt5a-stimulate non-canonical signaling, making use of the ability of the C-terminal fragment of DVl3 to act as a dominant-interfering peptide to the function of the same pathway (Figure 5D). Cells were transfected with empty vector (EV) or with the vector harboring the hDvl3-CT (497-716) of native sequence or hDvl3-CT with various substitutions. Whereas expression of the hDvl3-CT fragment sharply attenuates the signaling of the Wnt5a-stimulated NF-AT-dependent transcriptional response, the expression for the fragment in which residues 631-650 are deleted renders the dominant-interfering capability nil. Alanine-substitution of single His-repeat 637,638 likewise abolishes the ability of the hDvl3-CT to block non-canonical signaling. Similarly, the HH647,648AA mutant form of hDvl3-CT no longer functions as a dominant-interfering peptide with respect to the Wnt5a-stimulated non-canonical signaling pathway. Thus mutagenesis of His tandem repeats of either 637,638 or His 647,648 blocks dominant-interfering character of the expressed C-terminal fragment of Dvl3.

## Discussion

Although there is a myriad of molecules implicated in Wnt3a-sensitive β-catenin/Lef-Tcf-dependent transcriptional "canonical" signaling, Dishevelleds are common to all proposed models for the operation of this fundamental pathway in development [7,15,44,52,58-62]. These multivalent cytosolic phosphoprotein-scaffolds have been shown to play crucial roles in not only the canonical Wnt/β-catenin pathway [44,50,58], but as well as in planar cell polarity pathway [48,63]. Epistasis experiments reveal that Dishevelleds operate downstream of G-proteins in the fly [62] as well as in mammalian cells [7,34,63]. Whereas in the fly only a single Dishevelled (Dsh) is expressed, mammals express three isoforms of Dishevelleds, termed Dvl1, Dvl2, and Dvl3. Marked differences in native abundance distinguish the isoforms in this group, as do differences in their relative functional roles in enabling the "canonical" Wnt pathway [50]. Dvl3 is unique in many ways. The abundance of mammalian Dvl3 is very low (about 1/12-1/18 of Dvl2 or total Dvls) in many cell types including mouse F9, mouse P19 and human HEK[50]. Dvl3 is obligate for not only the Wnt "canonical" pathway, but also has been shown to be obligate for Wnt3a-stimulated p38 MAPK activation [64]. Furthermore, over-expression of Dvl3 itself stimulates Lef/Tcf-sensitive transcription, mimicking the effect of Wnt3a [50]. We explore the function of Dvl3 in the context of the Wnt5a-sensitive "non-canonical" pathway that operates through Frizzled2, G-proteins and their effectors (e.g. cGMP phosphodiesterase) and Ca^2+ ^mobilization, ultimately to regulate NF-AT-dependent transcription[34].

Mammalian Dvls are not functional redundant, but rather interdependent. All three Dvls are required for Wnt5a to stimulate non-canonical, NF-AT-sensitive transcriptional activation. Dvl2 constitutes > 90% of the total Dvl pool in F9 cells, whereas Dvl1 + Dvl3 sum to < 10%. Dvls display differential functional roles in the Wnt non-canonical pathway, similar to those reported earlier in Wnt3a-stimulated canonical signaling to β-catenin and Lef/Tcf-sensitive transcriptional activation [50]. The rank order of the Dvls with respect to overall function of the Wnt non-canonical pathway is identical to that for the canonical pathway. Notably, the siRNA-induced loss of Dvl3 individually, which results in a block of Wnt5a signaling to NF-AT-sensitive transcription, cannot be rescued by marked overexpression of either Dvl1or Dvl2. Only Dvl3 expression can rescue Wnt5a signaling in the Dvl3-deificient cells. Dvls isoforms are each obligate in canonical (i.e., Wnt3a/β-catenin/Lef-Tcf-sensitive transcription) and non-canonical (i.e., Wnt5a/Ca^2+^/NF-AT-sensitive transcription) signaling, but display important differences among the isoforms.

Mammalian Dishevelleds display a significant degree of sequence homology (> 70%). The highest homology in primary sequence is found in the conserved DIX, PDZ, and DEP domains; the least homology is found in Dvl3, especially when comparing the C-terminus among Dvls (Figure 6A). These observations focused our attention on the most unique region, the distal 200 amino acid residues extending from approximately residue 500 to the C-terminus (residue 716) in Dvl3. Deletion of this C-terminal region, like deletion of the DIX domain, blocked Dvl3 function in the Wnt non-canonical pathway. This same deleted fragment (hDvl3-CT) when expressed in cells displayed dominant-interfering character with respect to Wnt5a-stimulated activation of NF-AT-sensitive transcription. As regards to the importance of common DIX, PDZ and DEP domains of Dvl3 in Wnt non-canonical signaling, we found that DIX domain, like it is in canonical pathway [17,46], is essential for Dvl3 to function. On the contrary, PDZ domain, demonstrated indispensible for Dvl in Wnt canonical signaling [39], is non-essential for Wnt5a-sensitive non-canonical signaling. DEP domain, essential for Wnt-induced planar cell polarity [44,48], was found that deletion of it attenuates Dvl3 function in Wnt/NF-AT non-canonical pathway. We predict that the integrity of these common domains will be maintained in Dvl1 and Dvl2 for their functions in Wnt/NF-AT non-canonical signaling.

Our last goal focused on interrogating two unique and conserved landmarks in the Dvl3 C-terminus, namely single His-repeats at 637,638 and at 647,648. These single His-repeats are uniquely found in Dvl3 (not in Dvl1 and Dvl2); both C-terminal single His-repeats found in Dvl3 are conserved from frog (*Xenopus*) to human (*H. sapiens*), although lacking in the fly. Alanine-substitution of either single His-repeat or deletion of both simultaneously abolished the function of Dvl3. The functional loss of Dvl3 resulting from loss or mutation of these single His-repeats was demonstrated in two very different read-outs: loss of the ability of the full-length mutated hDvl3 to rescue Wnt5a-stimulated non-canonical signaling in Dvl3-deficient cells; and, loss of the dominant-interfering character of the hDvl3-CT fragment with respect to Wnt5a-stimulated non-canonical signaling. Thus, we report that Dvl3 is not functional redundant among mammalian Dvls with respect to the Wnt non-canonical signaling of Wnt5a. We demonstrate that overexpression of either Dvl1 or Dvl2 cannot rescue non-canonical signaling in cells made deficient of Dvl3 specifically. The unique structure of the distal region of Dvl3 C-terminus constitutes a major basis for the role of Dvl3 in the non-canonical pathway and this role is not shared for the Wnt3a-stimulated canonical pathway. Lastly, single His-repeats 637,638 and 647,648 are both structurally unique and functionally essential to Dvl3 in enabling Wnt5a-stimulated non-canonical signaling. Molecules that dock to and/or modify this short region containing two single His-repeats are prime targets for identification in the further interrogation of the molecular features of Wnt non-canonical signaling and the roles of Dvls.

## Conclusion

These data demonstrate through the use of several distinct strategies that His single amino acid repeats 637,638 and 647,648 in the distal C-terminus of Dvl3 are uniquely essential to function of the Wnt non-canonical pathway (Wnt5a > Frizzled-2 > cGMP phosphodiesterase/Ca^2+^-mobilization regulating the activation of NF-AT), yet non-essential to the function of the Wnt3a-stimulated canonical pathway (Wnt3a > Frizzled-1/LRP6 > β-catenin accumulation regulating the activation of Lef/Tcf-dependent transcription).

## Methods

### Cell culture and transfection

Mouse F9 teratocarcinoma cells were obtained from the ATCC collection (Manassas, VA). Cells were cultured in Dulbecco's modified Eagle's medium (Cellgro, Manassas, VA) supplemented with 15% heat-inactivated fetal bovine serum (Hyclone, South Logan, UT) at 37°C in a humidified atmosphere with 5%CO_2_. For transfection, plasmid DNA was introduced into cells by using LipofectAMINE and PLUS Reagent (Invitrogen) as per the manufacturer's instruction. Clones stably transfected with pcDNA3.1 harboring either rat Frizzled-2 (rFz2) or a chimera receptor of β_2_-adrenergic receptor/rat Frizzled-2 (β2AR/Fz2), were co-transfected with NF-AT-sensitive luciferase reporter gene (pNF-AT-Luc, Stratagene), according to the manufacturer's instruction. At least three stable clones were selected in medium containing G418 (0.4 mg/ml) and used in experiments.

### Construction of plasmids

Expression vector pcDNA3.1 harboring eGFP-tagged human Dvls (hDvl1-eGFP, or hDvl2-eGFP, or hDvl3-eGFP) was constructed as described [50]. Plasmid harboring Dapper1 was a kind gift from Dr. Ken-Ichi Takemaru (SUNY at Stony Brook). HA- or Myc-tagged (at N-terminus) human Dvls were generated by PCR amplification using pfu DNA polymerase, and PCR products were inserted into pCMV-HA or pCMV-Myc vector (BD Biosciences). Similar subcloning procedures were employed to generate HA- or Myc-tagged hDvl3 mutants truncated at the C-terminus (hDvl3ΔC), as well as Dvl3 C-terminal fragment (Dvl3-CT). To generate full-length hDvl3Δ631-650 or Dvl3-CTΔ631-650, plasmids harboring either hDvl3 or Dvl3-CT were amplified by PCR by using the following primers: 5'CCGAGCTACGGTCCTCCC3' and 5' CTCGCTGGCCGCCGGCC3'. Human Dvl3 and Dvl3-CT carrying point mutations were generated by using QuickChange Site-Directed Mutagenesis Kit (Stratagene), following the manufacturer's instructions. The authenticity of all constructs was confirmed by direct DNA sequencing.

### Knock-down of mouse Dvl by small interfering RNA (siRNA)

Cells were treated with 100 nM siRNA targeting one specific isoform of Dvl using Lipofectamine 2000 (Invitrogen) for 48 hours. For rescue experiments, cells were first treated with siRNA for 24 hours and then transfected with expression vector harboring a Dvl or Dvl mutant. Cells were cultured for additional 24-36 hours and then challenged by Wnt5a (50 ng/ml, R&D Systems). At least two sets of siRNA targeting each isoform of Dvl were used to ensure the avoidance of experimental results from the effects of off-target. Following siRNA pairs were used to target mouse Dvl1 (CCAGGAUAUUGGCUUGACAtt and UGUCAAGCCAAUAUCCUGGtt; CGUGAACAAGAUCACCUUUtt and AAAGGUGAUCUUCACGtt), mouse Dvl2 (GGAAGAGAUCUCCGAUGACtt and GUCAUCGGAGAUCUCUUCCtt; GGGUUGUCUCCUGGCUUGUtt and ACAAGCCAGGAGACAACCCtt) and mouse Dvl3 (GGAAGAGAUCUCGGACGACtt and GUCGUCCGAGAUCUCUUCCtt; GGAGAAUCUGGACAAUGACtt and GUCAUUGUCCAGAUUCUCC).

### Luciferase assay of NF-AT-sensitive transcriptional activation

Mouse F9 cells stably co-transfected with NF-AT reporter gene and expression vector harboring Fz1, or Fz2, or β2AR/Fz2 were cultured in 12-well plates. Cells were challenged with or without Wnt5a (50 ng/ml), Wnt3a (20 ng/ml) or isoproterenol (Iso, 10 μM) for 6 hour. Cells lysates were centrifuged, supernatants collected and luciferase activity measured as described [34].

### Immunoblotting

Cell lysates were subjected to SDS-polyacrylamide gel electrophoresis (SDS-PAGE) and the resolved proteins were transferred onto nitrocellulose membranes. The sources of primary antibodies are as follows. Anti-Dvl1 (3F12), anti-Dvl2 (10B5) and anti-Dvl3 (4D3): Santa Cruz Biotechnology; anti-Myc: Sigma-Aldrich; anti-eGFP: Clontech. Blots were washed and incubated with peroxidase-conjugated secondary antibodies as described [56]. The immune complexes were made visible by enhanced chemiluminescence method.

### Immunoprecipitation

Cells were lysed in a lysis buffer containing a cocktail of protease inhibitors as described [50]. Supernatant were collected after centrifugation (total lysates). Total lysates were incubated with protein A/G agarose coupled with anti-Dvl1, or -Dvl2 or -Dvl3 antibodies at 4°C and then subjected to centrifugation. Supernatants were collected (post-IP). Protein A/G agarose beads were washed 3 times with a buffer containing 20 mM Tris (pH8.0), 150 mM sodium chloride, 5 mM sodium ethylenediaminetetraacetate and 1% TritonX-100. Proteins precipitated (IP) were eluted in Laemmli's solution by boiling. Total lysates, post-IP and IP products were then subjected to immunoblotting analysis, as described above.

### Determination of intracellular cGMP accumulation

Intracellular cGMP concentration was determined by using a commercial cGMP ELISA kit (Cayman Chemical, Ann Arbor, MI), as previously described [34]. Briefly, mouse F9 cells stably expressing rFz2 were seeded onto 12-well plates and treated with control siRNA or siRNA specifically targeting one isoform of Dvl for 48 hours. Cells were stimulated with Wnt5a (50 ng/ml) for 45 min and lysed. Supernatants were collected by centrifugation and cGMP determined.

### Measurement of intracellular Ca^2+^

Cells were cultured on collagen-coated, 35-mm glass dishes (MatTek Corporation, Ashland, MA). The intracellular Ca^2+ ^was determined as described previously [34,56]. Briefly, cells were loaded with 2 μM Fura-2 acetoxymethyl ester (Molecular Probes, Inc.) in Krebs buffer composed of 128 mM NaCl, 5 mM KCl, 80 mM NaH_2_PO_4_, 1.3 mM MgSO_4_, and 1.3 mM CaCl_2 _for 40 minutes at 37°C. Excess Fura-2 was removed and cells were excited at 340 nm and 380 nm by using Lambda DG4 fast filter switcher. The intensity of emitted fluorescence monitored immediately after the administration of Wnt5a (50 ng/ml). Results were expressed as ratios of emission intensity excited at 340 nm over 380 nm (A_340_/A_380_).

### Indirect immunofluorescence microscopy

Mouse F9 cells stably expressing rFz2 were cultured on coverglass-chamber slides pre-coated with collagen. Cells were rinsed twice with an isotonic buffer and fixed in 3% paraformaldehyde containing 0.1% Triton 100. After incubation in the buffer containing 2% bovine serum albumin for 20 minutes, cells were washed and anti-Dvl2 antibody (1:1000, a kind gift from Dr. Mikhail V. Semenov, Harvard Medical School) was added. For double staining, cells were then incubated with anti-Dvl3 (1:200, from R&D Systems) at 37°C for 30 minutes. Following secondary antibodies were used: Alexa Fluor 594-conjugated donkey anti-goat IgG (1:1000) and Alexa Fluor 488-conjugated goat anti-rabbit IgG (1:1000). Fluorescence staining of Dvl2 and Dvl3 in cells was recorded by using a Zeiss LSM510 META confocal microscope in the Microscopy Imaging Center at SUNY at Stony Brook.

### Statistical analysis

All experiments were replicated at least three times. Within each experiment, assays were conducted at least in triplicate. Data are expressed as the means ± s.e.m. Comparisons of data among groups were performed with one-way analysis of variance followed by the Newman-Keuls test. Statistical significance (*p *value of less than 0.05) is denoted with an asterisk or a pound symbol.

## Abbreviations

cGMP: cyclic GMP; DMEM: Dulbecco's modified Eagle's medium; Dsh: Fly Dishevelled; Dvl1: Dishevelled-1; Dvl2: Dishevelled-2; Dvl3: Dishevelled-3; KD: knock-down; NF-AT: Nuclear Factor of Activated-T cells; rFz: rat Frizzled

## Competing interests

The authors declare that they have no competing interests.

## Authors' contributions

All authors contributed to the evolution of the study design; LM and YW contributed equally, performed the studies, gathered the data, and outlined a draft of the manuscript. HYW wrote the manuscript; all other authors edited the drafts, read and approved the final version of this unpublished work.

## Supplementary Material

Additional file 1 **Dvl1, Dvl2, and Dvl3 expression are each required for Wnt5a/Fz2/NF-AT signaling**. Mouse F9 cells stably co-transfected with pNF-AT-Luc and pcDNA3.β2AR/Fz2 were cultured in 12 well plates. Suppression of Dvl1, Dvl2, or Dvl3 individually was conducted by using either control siRNA or a second set of siRNA (as described in "Materials and Methods") targeting Dvl1, or Dvl2 or Dvl3, respectively. Suppression of Dvl1, 2 or 3 by using siRNA was examined by immunoblotting (top panel). The blots of actin were shown as loading controls. Cells were treated with vehicle (-Iso), or isoproterenol (+Iso, 10 μM) for 6 hr and the activity of NF-AT-dependent luciferase was measured (bottom panel). Click here for file
