# Supplementary material for: Dishevelled-3 C-terminal His single amino acid repeats are obligate for Wnt5a activation of non-canonical signaling
Source: J Mol Signal. 2010 Nov 23;5:19. doi: 10.1186/1750-2187-5-19 (PMC3003240; doi:10.1186/1750-2187-5-19)
Supplement: Additional file 1 — Dvl1, Dvl2, and Dvl3 expression are each required for Wnt5a/Fz2/NF-AT signaling. Mouse F9 cells stably co-transfected with pNF-AT-Luc and pcDNA3.β2AR/Fz2 were cultured in 12 well plates. Suppression of Dvl1, Dvl2, or Dvl3 individually was conducted by using either control siRNA or a second set of siRNA (as described in "Materials and Methods") targeting Dvl1, or Dvl2 or Dvl3, respectively. Suppression of Dvl1, 2 or 3 by using siRNA was examined by immunoblotting (top panel). The blots of actin were shown as loading controls. Cells were treated with vehicle (-Iso), or isoproterenol (+Iso, 10 μM) for 6 hr and the activity of NF-AT-dependent luciferase was measured (bottom panel). [file 1750-2187-5-19-S1.PDF]

Supplementary Data

Figure S1

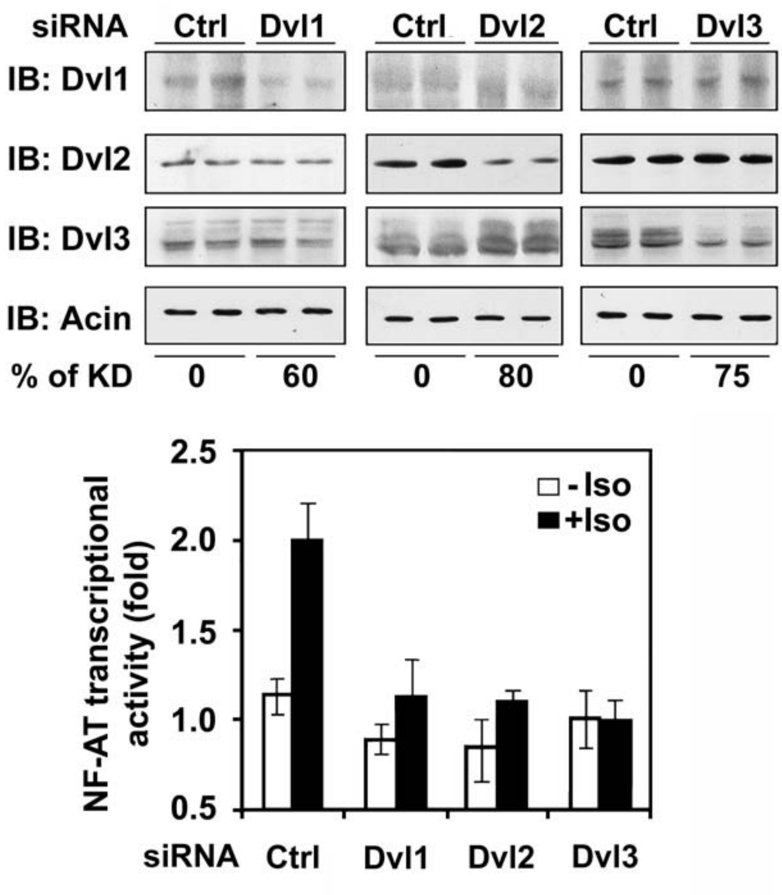

Supplementary Materials

Figure legends

Figure S1. Dvl1, Dvl2, and Dvl3 expression are each required for Wnt5a/Fz2/NF-AT signaling.

Mouse F9 cells stably co-transfected with pNF-AT-Luc and pcDNA3.β2AR/Fz2 were cultured in 12 well plates. Suppression of Dvl1, Dvl2, or Dvl3 individually was conducted by using either control siRNA or siRNA targeting Dvl1, or Dvl2 or Dvl3 respectively (a second set of siRNA as described in "Materials and Methods"). Suppression of Dvl1, 2 or 3 by using siRNA was examined by immunoblotting (top panel). The blots of actin were shown as loading controls. Cells were treated with vehicle (-Iso), or isoproterenol (+Iso, 10 μM) for 6 hr and the activity of NF-AT-dependent luciferase was measured (upper panel).
